# Gold-Enhanced Multielectrode Electrochemical Immunosensor for Rapid Electrochemical Detection of Dengue NS1

**DOI:** 10.1021/acsomega.6c04898

**Published:** 2026-10-19

**Authors:** Luiz Otávio Orzari, Bruna Silva Faria, Jéssica Rocha Camargo, Lucas Aguilera Mariano, Déborah Christine Azzi, Alexandre Gatti, Geisiany Maria de Queiroz, Bruno Campos Janegitz

**Affiliations:** † Institute of Chemistry, State University of Campinas, Campinas, São Paulo 13083-970, Brazil; ‡ Center for Innovation in New Energies, Campinas, São Paulo 13083-841, Brazil; § Laboratory of Sensors, Nanomedicine and Nanostructured Materials, 67828 Federal University of São Carlos, Araras, São Paulo 13600-970, Brazil; ∥ Department of Nature Sciences, Mathematics and Education, Federal University of São Carlos, Araras, São Paulo 13600-970, Brazil; ⊥ 648561 Faculdade São Leopoldo Mandic, Araras, São Paulo 13606-134, Brazil; # ADB Pesquisa e Desenvolvimento Ltda, Araras, São Paulo 13600-001, Brazil

## Abstract

Dengue is one of the most widespread viral diseases in the world, transmitted mainly by *Aedes aegypti* and *Aedes albopictus*. In tropical and resource-limited regions, rapid and accurate diagnostic tools are of utmost importance, especially when aiming for effective control, but current detection methods face limitations in sensitivity, specificity, or accessibility. In this work, a multielectrode disposable electrochemical immunosensor was developed for the detection of the nonstructural protein 1 (NS1), an early biomarker of dengue infection. This device presents four fully individual and functional working electrodes modified with gold nanoparticles. This configuration enables replicate measurements and improved reproducibility within a single platform while using minimal sample volume. The electrodeposition of Au nanoparticles was optimized through Design of Experiments approaches, allowing the system to suitably determine the target protein with a specific biorecognition architecture based on antibody–antigen recognition. This biosensor was characterized by morphological and electrochemical techniques, achieving a linear response between 1.0 and 200 ng mL^–1^ and a limit of detection of 0.34 ng mL^–1^. Afterward, human serum samples spiked with NS1 showed recovery rates between 96.6% and 115%. All results indicate that the proposed multielectrode immunosensor is a promising, label-free, and portable diagnostic alternative for NS1 determination while maintaining a relatively low cost and simple preparation.

## Graphical abstract

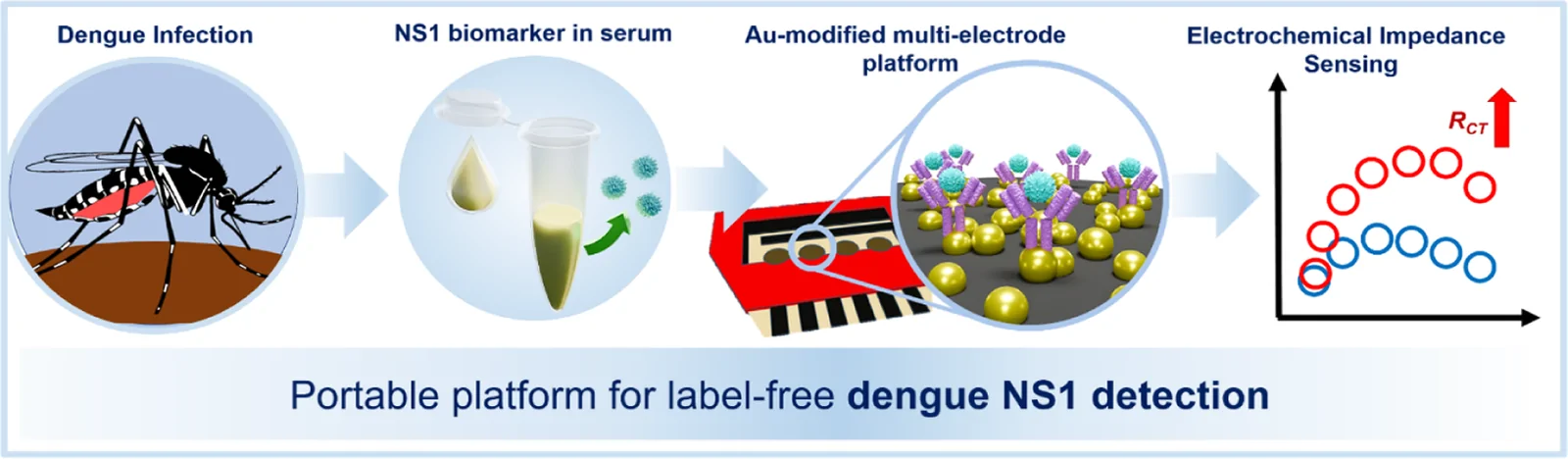

## 1 Introduction

Being primarily transmitted by *Aedes aegypti* and *Aedes albopictus* mosquitoes, the dengue virus is currently responsible for the most common viral disease worldwide. This Flavivirus (DENGV) consists of four separate serotypes: DENV1, DENV2, DENV3, and DENV4, all of which originated from wild strains in Southern Asia.^1–4^ With an increase in its expansion in the Northern Hemisphere nowadays, the virus began its spread in tropical and subtropical regions in 50-year intervals, and now can be found everywhere.^1–4^ Through the saliva of contaminated female mosquitoes, the DENGV can rapidly infect a local population: after 5 to 8 days of incubation, the disease commonly begins with a severe headache and intense fever, due to dehydration. Afterward, it can become fatal, potentially evolving to hemorrhagic fever or dengue shock syndrome in a few hours if not treated properly.^1–4^

Many methodologies, in recent decades, have been proposed for the determination of DENGV; however, applying such devices in industry and healthcare remains a challenge.^5–7^ Most of the available options are based on virus isolation, serological tests, or similarly complicated approaches, requiring significant effort, time, and resources.^8,9^ As an alternative, several biomarkers have been explored for dengue diagnostics, including viral RNA,^10,11^ viral antigens,^12,13^ and host antibodies.^14^ Among these, the nonstructural protein 1 (NS1) has emerged as one of the most clinically relevant targets, mostly due to its reliability: it is secreted in the bloodstream from the early stages of the infection, even within the first day of fever.^15–17^ Normally, antibody-based devices require the development of the disease to be able to achieve an optimal response range. However, NS1-based ones can provide a much shorter diagnostic window, coinciding with the acute phase, making it an attractive route for quick interventions.^9,18,19^

The most prominently employed methods for NS1 detection are ELISA and rapid diagnostic tests, but they also present important limitations: ELISA requires specific laboratory infrastructure, trained personnel, and long assay times in order to achieve its high sensitivity and specificity.^20,21^ Rapid tests, on the other hand, are quite fast and easier to operate, but suffer from lesser sensitivity and specificity, being remarkably inconsistent in the cases of secondary infections.^22,23^ These highlight the need for new diagnostic technologies that combine accuracy, speed and ease of operation, and portability.

Electrochemical systems appear as one of the alternatives for NS1 detection, which contemplates all required attributes, especially when considering the advent of multielectrode platforms.^24^ Multielectrode configurations are particularly advantageous when the goal is not only multianalyte detection but also the acquisition of replicate signals within the same device.^25^ This approach improves repeatability and reproducibility while reducing sample consumption, which is especially important for point-of-care testing and clinical samples available in limited volumes.^26^ With intelligent treatments or modifications, such as the addition of specific recognition structures, it is possible to analyze different biomarkers in the same time frame, or collect multiple signals from the same one, increasing the reliability of the device many times over.^27,28^ In these aspects, lab-made conductive inks can be used to manufacture this type of device quickly, simply, and at a relatively low cost.^29–31^ Modifying electrodes with electrochemically active materials, such as gold particles, can increase their electroactive area, facilitate electron transfer, and even allow the immobilization of antibodies, increasing sensitivity, conferring selectivity, and reducing detection limits.^32–34^

Furthermore, another important aspect of these electrochemical biosensors should be noted: the use of relatively low-cost materials in their production can increase their availability and the accessibility of the clinical diagnostics they provide. Arthropod-borne viral diseases such as dengue, Zika, and chikungunya have been prevalent in resource-poor regions of the globe for more than five decades, making them prime examples of neglected tropical diseases (NTDs). The “neglected” classification is due to the lack of attention they receive from the pharmaceutical industry and from disease prevention efforts by public policymakers, despite their high mortality rates.^35–37^ Many countries affected by these viruses cannot afford to adequately treat their populations, making these detection systems attractive resources for advancing NTD elimination.

In this context, we propose the development of a multielectrode screen-printed electrochemical immunosensor with four individually functional electrodes for NS1 detection, designed to enable replicate measurements and improved analytical reliability within a single device using small sample volumes. These biosensors are especially attractive for point-of-care applications, since they combine portability with affordability, while maintaining the sensitivity required for clinical diagnostics.

## 2 Experimental Section

All reagents used in this work were of analytical grade, obtained from Sigma-Aldrich/Merck, Fluka, and/or Fisher Chemical. Ultrapure water (Millipore Synergy system, resistivity ≥ 18.2 MΩ cm) was used to prepare all solutions. Tetrachloroauric acid was obtained from Aldrich. For electrochemical analyses, a potentiostat/galvanostat PGSTAT204 Metrohm (Eco Chemie) was employed, managed by the Nova 2.1.5 software. Such analyses were carried out with a six-electrode system, with four disposable working electrodes, made of conductive paste Carboink (ADB Sensores LTDA, Brazil) and goldnanoparticles. The auxiliary and pseudoreference electrodes were made of the same carbon material. The masks made of adhesive paper (Colacril), applied for the production of disposable electrodes, were developed using the Silhouette Studio software, with the Silhouette Cameo 3 cutting printer being used to produce them. Scanning electron microscopy (SEM) images were obtained by a ThermoFisher Scientific Prisma E Scanning Electron Microscope, at 5 kV accelerating voltage and 50 Pa low vacuum mode. For the electrical characterization, the Kaivo Four-Point Probe Resistance Tester (DC) (Guangdong, China) was used.

### 2.1 Multielectrode Disposable Immunosensors Preparation

The conductive paste Carboink was commercially purchased from ADB Research and Development Ltd.a. Initially, an acetate sheet was used as the substrate (Figure 1, step I). Then, the masks were adhered to this substrate (Figure 1, step II), and the conductive ink was spread using a spatula (Figure 1, step III). After application, the masks were removed (Figure 1, step IV), and the sensors were allowed to dry for at least 1 h before use (Figure 1, step V). Next, the delimiting mask was attached, the edges were trimmed to ensure proper fitting into the connector, and the Carboink SPE sensor was ready for use (Figure 1, step VI).

**Figure 1:**
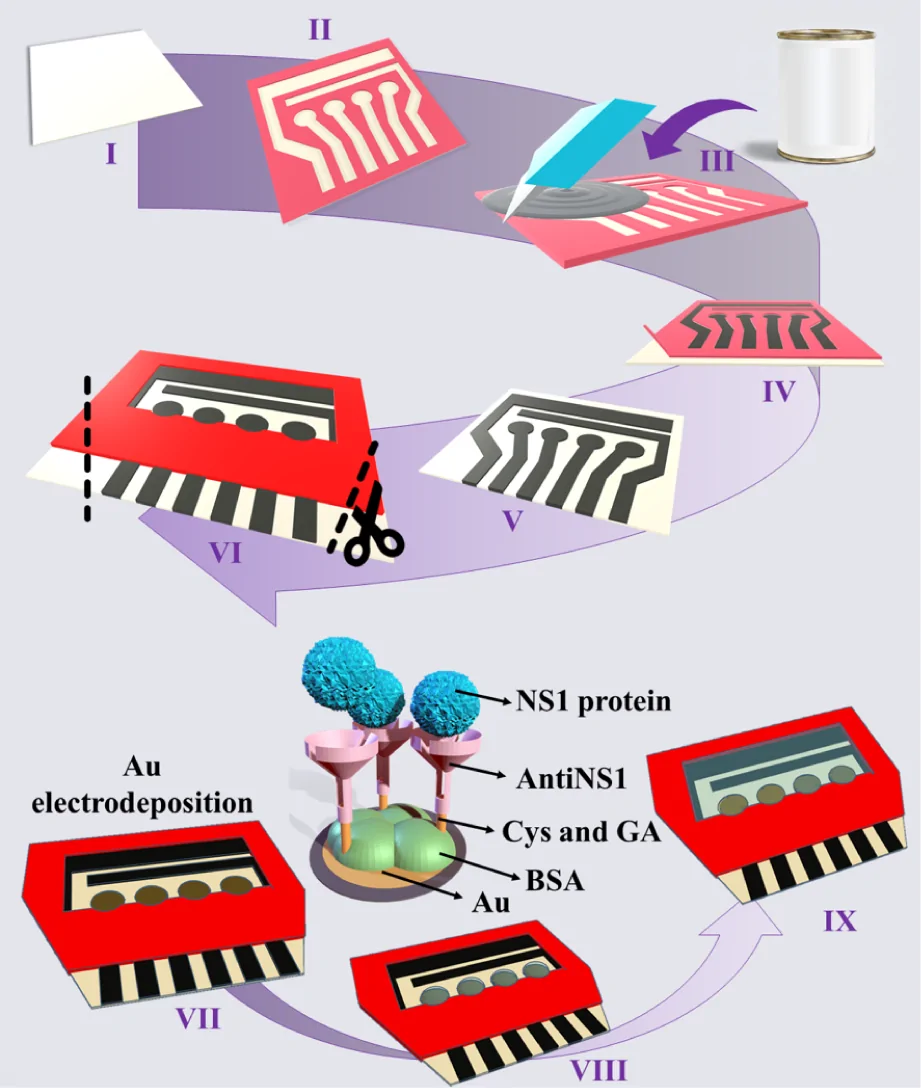
Preparation of disposable electrodes and immunosensor assembly. Steps I–VI: acetate sheet used as substrate (I); masks adhered to the substrate (II); conductive ink spread with a spatula (III); masks removed (IV); sensors dried for at least 1 h (V); delimiting mask attached and edges trimmed for connector fitting (VI). Steps VII–IX: gold electrodeposited on the electrode surface (VII); biomodification solutions applied for the optimized reaction time (VIII); immunosensor finalized and ready for use (IX).

For the construction of the immunosensor, gold was first electrodeposited onto the electrode surface (Figure 1, step VII). Subsequently, the solutions required for biosensor assembly were applied to the working electrodes and allowed to react for the previously optimized time (Figure 1, step VIII). Finally, the resulting biosensor was ready for use (Figure 1, step IX). A step-by-step demonstration video of the immunosensor fabrication process is provided in the Supporting Information (Video 1).

For the selectivity study, the same experimental procedure was employed; however, the NS1 solution was replaced by a SARS-CoV-2 spike protein solution at the same concentration (150 ng mL^–1^). After incubation, the electrodes were rinsed with phosphate buffer and subjected to electrochemical impedance measurements under identical conditions, allowing a direct comparison between the target analyte and the potential interfering protein.

Au was electrodeposited over the working electrodes, and the procedure was optimized with the aid of Design of Experiments matrices. Chronoamperometry was investigated with regard to the deposition time and deposition potential. Gold precursor solution (HAuCl_4_·3H_2_O, Sigma-Aldrich, purity ≥ 99.9%) was used for electrodeposition. In order for the devices to detect the NS1 protein, further modifications were necessary, as explored by the literature.^38–43^ The method employed uses 2.0 μL of cysteamine (CYS, 10 mmol L^–1^) and 2.0 μL of glutaraldehyde (GA, 2.5% (v/v)) to add interactive sites over the working surface, to covalently bond with AntiNS1. The CYS/GA strategy was selected due to its simplicity, rapid formation of stable imine bonds, and suitability for porous carbon-based substrates. Although EDC/NHS chemistry provides more controlled amide coupling, GA cross-linking is widely reported as a robust alternative for immobilizing antibodies on nanostructured gold surfaces, especially in disposable platforms where simplicity and reproducibility are required.^44^

After the addition of the antibody, obtained from a commercial source (Sino Biological Inc., Beijing, China; catalog no. 40263-MM02, mouse monoclonal antibody, IgG1, clone 02, anti-NS1), 2.0 μL of a 1.0 mg mL^–1^ solution was applied onto the electrode surface. Subsequently, a 1.0% (w/v) bovine serum albumin (BSA) solution was added to block nonspecific binding sites.

Dengue virus nonstructural protein 1 (NS1) was obtained as a recombinant protein (Sino Biological Inc., Beijing, China; catalog no. 40263-V07H), expressed in HEK293 cells with a polyhistidine tag and purity higher than 90%. The stock solution (1 g L^–1^) was prepared according to the manufacturer’s instructions and stored under recommended conditions. Working solutions were prepared by dilution in phosphate buffer (0.1 mol L^–1^, pH 7.4) to obtain concentrations ranging from 1.0 to 200 ng mL^–1^. Then, 2.0 μL of NS1 protein solution was added to the electrode surface to allow the specific antigen–antibody interaction, completing the immunosensor assembly. Each modification step was carried out for 1 h, except for the BSA blocking step, which was performed for 10 min. After each step, the electrodes were rinsed with a 1.0 mmol L^–1^ phosphate buffer (PB, pH = 7.4) solution to eliminate excess molecules that did not take part in the self-layer formation. By investigating the charge transfer resistance (R_CT_) variation of a faradaic process at the last step, it was possible to calibrate the device for such a target. A ferrocenemethanol solution, at 1.0 mmol L^–1^, was used for such a study, in all necessary electrochemical techniques.

For the stability and reproducibility studies, the immunosensors were fabricated up to the BSA blocking step (BSA/AntiNS1/GA/CYS/AuCB) and subsequently stored under refrigerated conditions (4.0 °C). During the storage period, the devices were maintained slightly moistened with phosphate buffer solution (1.0 mmol L^–1^, pH 7.4) to preserve the biological activity of the immobilized antibody and avoid dehydration of the sensing interface. Before each daily measurement, a fresh aliquot (2.0 μL) of the NS1 solution (150 ng mL^–1^) was added onto the electrode surface and allowed to interact with the immobilized antibodies under the same conditions employed during calibration. This procedure was intentionally adopted to mimic the practical use of the immunosensor in real sample analysis, where antigen recognition occurs immediately before measurement. After the antigen–antibody interaction step, the electrodes were rinsed with phosphate buffer to remove unbound species and electrochemical impedance measurements were performed. Four independently prepared immunosensors were evaluated over five consecutive days following the same protocol.

### 2.2 Electrodeposition Optimization

The electrodeposition of Au was investigated with the aid of a circumscribed central composite design, aiming to obtain the most effective parameter values and their combination. This experimental design is an expansion of common linear designs, like Box-Behnken, but modified to support second-order interactions.^45,46^ In it, each variable is studied in 5 levels, as can be seen in Table 1, and the experimental distribution of this design allows constant variability predictions at all points, as well as the calculation of linear and quadratic responses with few experiments.^45,46^ In this study, the Au electrodeposition time was the first variable investigated at 30 and 300 s (−1 and +1, respectively), while the potential applied was evaluated at −0.9 and −0.6 V (also −1 and +1, respectively). After deposition, cyclic voltammetry was used to obtain the mean current values correlated to the metallic peak. Afterward, each variable and interaction effect were calculated as explained by eq 1:

1 $$Effect=\frac{\left({\overset{―}{vi}}_{-1}\right)-\left({\overset{―}{vi}}_{+1}\right)}{2}$$

where ${\overset{―}{vi}}_{-1}$ is the average response when the variable *i* is at −1, while ${\overset{―}{vi}}_{+1}$ is the average value of the responses when *i* is +1. When dividing the effect by 2 once more, the coefficient associated with each variable is obtained and a mathematical model is then achieved.

**Table 1:** Central Composite Design Matrix for Au Electrodeposition (Coded Values) and Response Obtained^a^

Exp.	M	v1	v2	11	22	12	I_Mean (mA)_
1	1	–1.0	–1.0	1.0	1.0	1.0	23.3
2	1	–1.0	1.0	1.0	1.0	–1.0	17.5
3	1	1.0	–1.0	1.0	1.0	–1.0	59.0
4	1	1.0	1.0	1.0	1.0	1.0	104
5	1	–1.03	0.0	1.08	0.0	0.0	17.3
6	1	1.40	0.0	1.98	0.0	0.0	115
7	1	0.0	–1.67	0.0	2.78	0.0	13.3
8	1	0.0	1.67	0.0	2.78	0.0	46.7
CP1	1	0.0	0.0	0.0	0.0	0.0	3.75
CP2	1	0.0	0.0	0.0	0.0	0.0	36.0
CP3	1	0.0	0.0	0.0	0.0	0.0	20.3

a **Exp.:** Experiment index; **M:** matrix mean column; **v** *i*: variable *i*; ***ii***: quadratic effect of variable *i*; **CP#:** central point of index #; **I**_**mean**_: mean values of current peak responses.

### 2.3 Sample Preparation

NS1 protein solutions were freshly prepared in phosphate buffer (0.1 mol L^–1^, pH 7.4). Human serum samples (from male AB clotted whole blood, Lot #SLBV1669) was purchased from Sigma-Aldrich (USA) and diluted (1:10) before analysis. Blank samples (without NS1) were used to evaluate baseline impedance response.

### 2.4 Selectivity, Storage Stability, and Contact Angle Measurements

The selectivity of the developed immunosensor was evaluated by investigating the influence of common endogenous electroactive compounds naturally present in human serum and a nontarget viral protein. Glucose (3.0 × 10^–2^ mol L^–1^, 5.40 mg mL^–1^), dopamine (1.0 × 10^–4^ mol L^–1^, 0.015 mg mL^–1^), and SARS-CoV-2 spike protein (150 ng mL^–1^) were individually evaluated under the same experimental conditions employed for Dengue NS1 detection. Electrochemical impedance spectroscopy (EIS) measurements were performed in 1.0 mmol L^–1^ ferrocenemethanol prepared in 1.0 mmol L^–1^ phosphate buffer (pH 7.4), using an applied potential of 75 mV.

The storage stability of the immunosensor was evaluated by storing the modified electrodes (BSA/AntiNS1/GA/CYS/Au/CB) at 4 °C. Short-term stability was assessed over five consecutive days, while long-term storage stability was investigated after 30 days of refrigerated storage. In both cases, the NS1 antigen (150 ng mL^–1^) was incubated immediately before the electrochemical measurements, and the impedance responses were compared with those of freshly prepared immunosensors.

Contact angle measurements were performed to evaluate the wettability of the electrode surface after each modification step. A 2 μL droplet of ultrapure water was deposited onto the electrode surface, and images were acquired using an iPhone 13 Pro coupled to a custom-built contact angle measurement system described in ref 47 The contact angle values were determined from the acquired images using Blender software by analyzing the droplet profile.

## 3 Results and Discussion

### 3.1 Morphological and Electrochemical Characterization

The addition of metal particles to the surface of an electrode has attracted considerable scientific attention in recent years due to the numerous possibilities surrounding the topic. Various aspects of this deposition can be modified and explored to obtain defined particle shapes or properties that can enhance the application of the work or even provide essential information. In this work, we opted for electrodeposition with potentiostatic chronoamperometry, and the technique parameters, deposition time (v1) and applied potential (v2) were studied with a central composite design experimental matrix, found in Table 1. v1 was investigated at the values of 30 and 300 s (−1 and +1, respectively), while v2 was tested at the values of −0.9 to −0.6 V (also respectively −1 and +1). The experimental variability was estimated from the triplicate measurements and central point replicates. Cyclic voltammograms in the presence of KCl, 0.1 mol L^–1^, obtained by the modified devices, at a scan rate (v) of 50 mV s^–1^, were carried out to investigate the deposition. Information on the anodic and cathodic peak currents of the Au oxide formation and reduction process was used to better understand the Au surface coverage by this method, and the mean value between the absolute currents was used in subsequent analyses.

By statistically analyzing the data, the following equation can be obtained:

2 $$I_{Mean}\left(mA\right)=20.7+\left(28.3\times v1\right)+\left(9.95\times v2\right)+\left(39.6\times v1^{2}\right)+\left(10.9\times v2^{2}\right)+\left(12.8\times v1\times v2\right)$$

in which it is possible to note that v1 showed a substantially stronger influence on the response than v2. Overall behavior of the mathematical model is also a quadratic one, as can be observed in Figure 2. Both parameters present a positive behavior to the system, with values close to the maximum studied (+1), which contributes to obtaining a higher response. The authors opted to define the following values as optimal: v1 = 300 s and v2 = −0.8 V, due to these being inside the experimental range and confidence, and still being closer to the extrapolated optimal value (red zone). Table S1 summarizes this optimization protocol.

**Figure 2:**
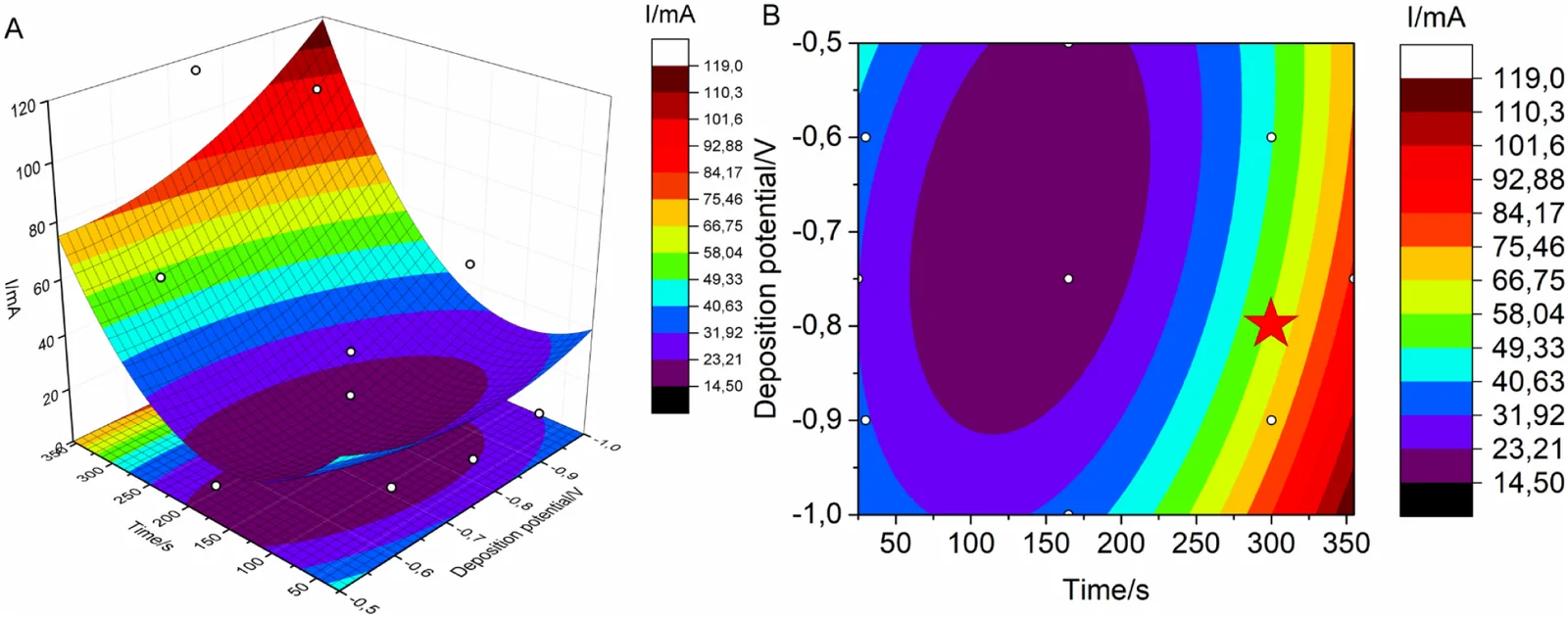
(A) Response surface and (B) contour graph for the Au electrodeposition experimental design. The red star indicates the defined optimal values.

With the Au deposition optimization completed, it was possible to characterize the device, seeking to better understand how the modification affected it. To achieve this, the techniques of scanning electron microscopy (SEM), X-ray Diffraction (XRD), Fourier-Transform Infrared Spectroscopy (FT-IR), cyclic voltammetry (CV), electrochemical impedance spectroscopy (EIS), contact angle, and four-terminal electrical resistance measurements were carried out.

The SEM micrographs of the conductive ink before gold electrodeposition (Figure 3A) display a heterogeneous and highly porous morphology, comprising interconnected carbon domains, binder regions, and voids, which are typical of conductive inks. This porous architecture is advantageous for electrochemical applications, as it enhances the electroactive area and electrolyte accessibility.^48^ ColorSEM mapping (Figure 3B) confirms that the surface is predominantly composed of carbon (C) and oxygen (O), with silicon (Si) attributed to the substrate or formulation fillers. No gold signal is detected, confirming the absence of intrinsic metallic species and indicating that charge transport occurs exclusively through the carbon network.

**Figure 3:**
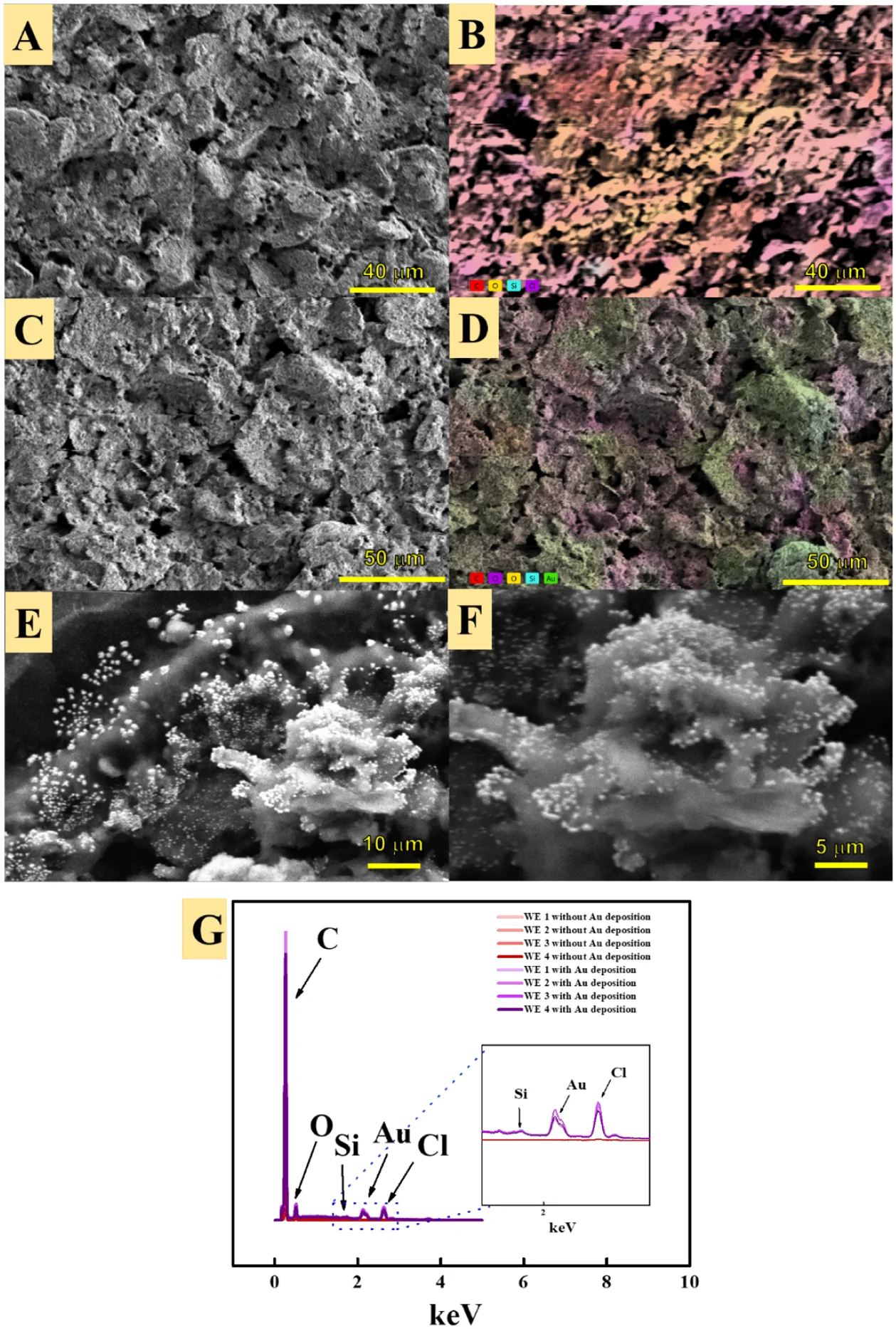
Morphological and elemental characterization of the carbon electrode before and after Au electrodeposition. (A) SEM image of the nonmodified electrode showing a porous carbon-based morphology. (B) ColorSEM mapping confirming the distribution of C, O, and Si without Au. (C) SEM image after Au deposition, preserving the porous architecture with localized metallic clusters. (D) ColorSEM mapping highlighting the spatial distribution of Au across the surface. (E) SEM image after Au deposition at 2500× magnification. (F) SEM image after Au deposition at 5000× magnification. (G) EDS spectra demonstrating the appearance of characteristic Au peaks (≈2.1–2.3 keV) only after electrodeposition. Also, we observe clearly gold nanoparticles on the carbon surface.

After electrodeposition, the Au-modified electrode (Figure 3C) preserves the original rough and porous morphology, indicating that the electrodeposition process does not alter the bulk structure of the conductive ink. Instead, subtle morphological changes associated with metallic cluster formation can be observed. ColorSEM mapping (Figure 3D) clearly reveals Au-rich regions homogeneously distributed over the electrode surface, demonstrating the successful incorporation of gold throughout the carbon matrix despite its inherent surface irregularity.

Higher-magnification SEM images (Figure 3E and 3F) provide direct evidence of the deposited gold nanostructures. Numerous bright nanosized particles are observed decorating the carbon surface and pore walls, forming uniformly distributed clusters rather than a continuous metallic film. This morphology is particularly advantageous because it substantially increases the density of electroactive sites while maintaining the porous architecture and accessibility of the carbon substrate. Such a nanostructured Au layer is expected to promote faster electron-transfer kinetics and provide a larger effective surface area for subsequent antibody immobilization.

The EDS spectrum (Figure 3G) further corroborates these observations. While carbon and oxygen remain the predominant elements, characteristic Au peaks appear in the 2.0–2.5 keV region, confirming successful gold deposition. The persistence of the intense carbon signal indicates that the deposited Au forms a discontinuous surface nanostructure rather than a thick metallic coating.^49^ Quantitative EDS analysis revealed an average Au content of 29.7 ± 4.31 wt.%, demonstrating reproducible and effective electrodeposition across the fabricated electrodes.

Overall, the SEM, ColorSEM, and EDS results demonstrate that the optimized electrodeposition protocol produces homogeneously distributed Au nanoclusters on the porous carbon electrode without compromising its structural integrity. This hierarchical micro/nanostructure is expected to enhance the electroactive surface area, improve charge-transfer properties, and provide a suitable platform for the immobilization of biomolecular recognition elements.

The X-ray diffraction pattern of the conductive ink (Cu Kα radiation, scanned from 2 to 90° in 2θ with a step size of 0.02°) indicates that the material is predominantly amorphous, with only minor crystalline contributions, see Figure 4. A broad halo between approximately 15 and 30° is observed, which is characteristic of amorphous materials and consistent with the presence of the alkyd resin and the amorphous carbon fraction in the carbonaceous dispersion.^50,51^ Superimposed on this background, a strong and relatively sharp peak appears at about 27°, attributed to the (002) plane of graphitic structures, indicating the existence of partially ordered carbon domains within the amorphous matrix.^51,52^ In addition, low-intensity secondary peaks are detected at approximately 19°, 22°, 24°, 30°, 36°, and 55°, which may be associated with small amounts of crystalline additives in the formulation or structural heterogeneities within the carbonaceous phase, such as semicrystalline regions or higher-order reflections from graphitic stacking.^50,52,53^ Overall, the results confirm that the ink is largely amorphous but contains graphitic domains that likely contribute to its electrical conductivity. This combination of amorphous and graphitic features is consistent with the ink’s composition, which includes a carbonaceous dispersion, alkyd resin, organic solvents, and additives.

**Figure 4:**
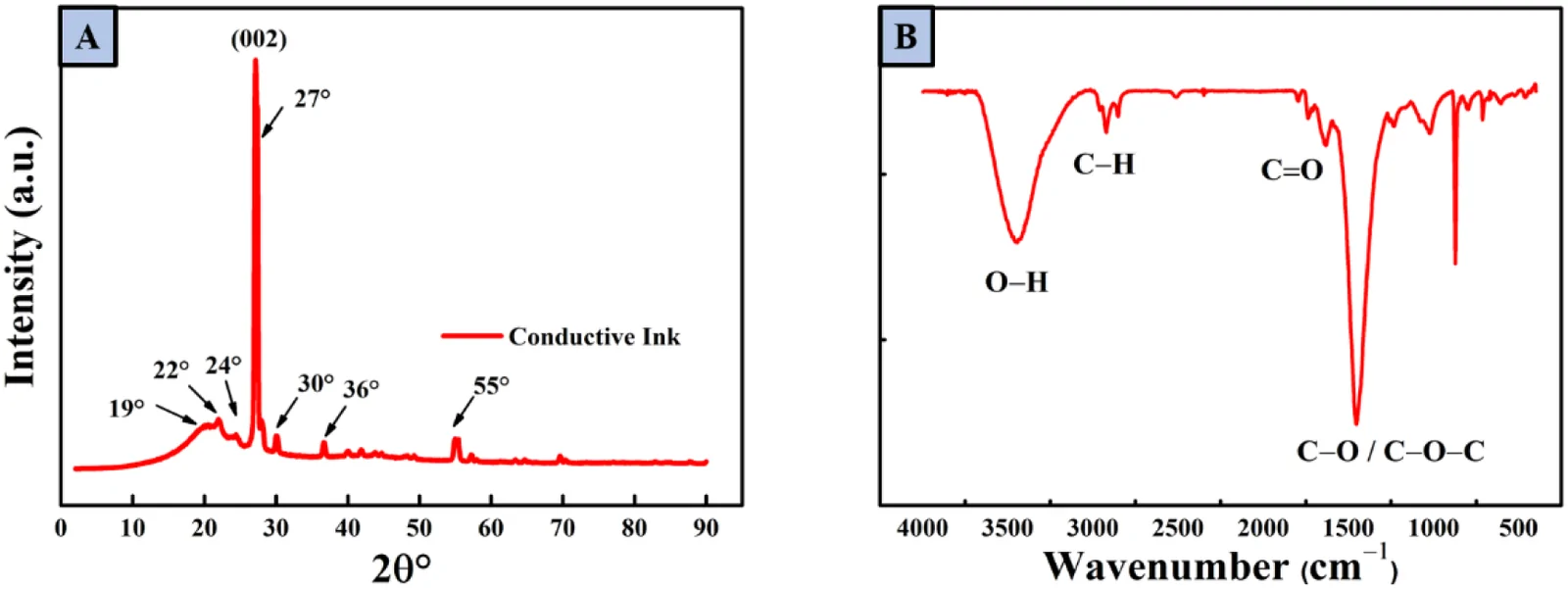
Morphological characterization of the conductive ink. (A) XRD pattern of the commercial conductive ink. (B) FT-IR spectrum of the commercial conductive ink obtained in KBr mode (4 cm^–1^ resolution, 64 scans).

The FT-IR spectrum of the conductive ink reveals the characteristic vibrational features of an alkyd-based polymeric matrix together with organic components typically present in coating formulations. A pronounced absorption band centered at approximately 1730 cm^–1^ corresponds to the ester C=O stretching mode, confirming the polyester backbone structure of the alkyd resin.^54^ The strong bands observed in the 2920–2850 cm^–1^ region are attributed to the asymmetric and symmetric stretching vibrations of aliphatic −CH_2_ and −CH_3_ groups, which originate from both the alkyd resin and residual organic solvents.^54^ In the fingerprint region, several intense absorptions between 1300 and 1000 cm^–1^ are assigned to C–O and C–O–C stretching vibrations of ester and ether functionalities, further supporting the predominance of polyester-type linkages.^55,56^ Additional bands near 1600 cm^–1^ and in the 750–700 cm^–1^ range indicate aromatic ring vibrations, consistent with phthalate-derived structural units commonly incorporated into alkyd resins.^54,56^ A broad feature around 3400 cm^–1^ suggests the presence of hydroxyl groups, which may arise from residual solvent molecules, terminal alcohol groups of the resin, or adsorbed moisture.^57^ As expected for carbon-black- or graphite-based dispersions, no distinctive infrared absorption bands attributable to the conductive carbonaceous phase are detected due to the low IR activity of highly conjugated carbon materials. Overall, the spectrum confirms that the ink formulation is dominated by an alkyd polyester network, accompanied by aliphatic and aromatic organic components and typical additive-derived functionalities.

The four-terminal resistance measurements revealed that the electrical resistance of the proposed device is 7.0 ± 0.6 Ω, which is considerably lower than commercial devices, as reported in the literature.^58^ Essays to investigate wettability were carried out with a droplet of water over each electrode, before and after modification, as can be observed in Figure 5A and B. Without modification, the contact angle was of ∼131°, while after the modification, this value changed to ∼103°, indicating that the device became more hydrophilic. However, due to the larger number (>90°), both devices were still considered to be hydrophobic.

**Figure 5:**
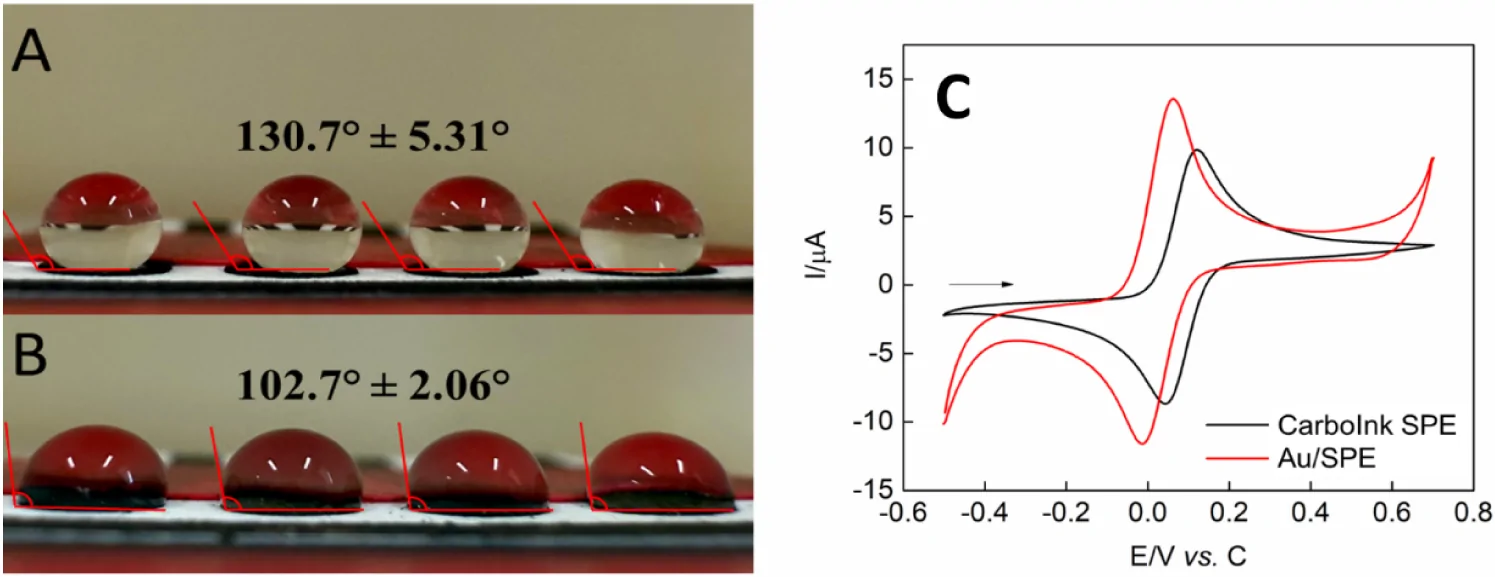
Contact angle images for (A) CarboInk SPE and (B) Au/SPE; (C) cyclic voltammograms obtained by CarboInk SPE **(black)** and Au/SPE **(red)** in the presence of 1.0 mmol L^–1^ ferrocenemethanol and 0.1 mol L^–1^ KCl; v = 50 mV s^–1^.

The electrochemical profile of the system was investigated by CV, in the potential window of −0.5 to 0.7 V, in the presence of 1.0 mmol L^–1^ ferrocenemethanol and 0.1 mol L^–1^ KCl, at 50 mV s^–1^, as can be seen in **Error! Reference source not found.**C for both modified and unmodified devices.

As can be observed, The SPE presented a redox pair at 122 (I_Anodic_ = 9.85 μA) and 42 mV (I_Cathodic_ = −8.67 μA), with a peak-to-peak separation (ΔE) of 80 mV and quotient between currents (I_Anodic_/I_Cathodic_) of 1.14, conferring a characteristic reversible behavior to this system, very close to theoretical values.^59^ With the addition of Au particles over the surface, the device behavior significantly changed: both anodic and cathodic peaks shifted toward more negative values: E_Anodic_ = 64 mV and E_Cathodic_ = −14 mV. The magnitude of the peaks obtained also increased significantly, with values of 13.6 and −11.6 μA, for anodic and cathodic peaks, respectively. The ΔE of the modified system slightly diminished to 78 mV, while the new calculated I_Anodic_/I_Cathodic_ is 1.17, indicating that, although the peaks are fairly impacted, the reversibility of the system is not. However, since the redox pair is found at more negative potential, it is safe to say that the presence of Au caused electrocatalytic effects in this system.

By varying the v between 10 and 150 mV s^–1^ for both modified and unmodified devices, it was possible to understand the electrochemical profile of such platforms. As can be observed in both devices presented a fairly linear behavior, with the average R^2^ for SPE being 0.9983, and for Au/SPE being 0.9952. These values indicate that the mass transfer occurs predominantly by diffusion. With the aid of the Randles-Ševčík equation for reversible systems,^60^ the data were used to estimate the device’s electroactive area. The value calculated for SPE was 0.067 ± 0.0016 cm^2^, while the value calculated for the Au-modified device was 0.109 ± 0.035 cm^2^. This indicates that the addition of Au particles over the surface is also responsible for an increment of active area, which could be beneficial to achieve higher sensitivity, see Figure S1.

Another technique that helps in the understanding of the system is the electrochemical impedance spectroscopy. This technique can confer information regarding the system charge and other physicochemical properties of the surface. The experiment was carried out using an applied potential of 5.0 mV, in the presence of 1.0 mmol L^–1^ ferrocenemethanol, in 0.1 mol L^–1^ KCl. Figure S2A shows the Nyquist diagram obtained for the CarboInk SPE in the presence of 1.0 mmol L^–1^ ferrocenemethanol in 0.1 mol L^–1^ KCl. The experimental data were fitted using the equivalent circuit shown in the inset (x^2^ = 2.77 × 10^–4^), which is consistent with a Randles-type model incorporating a Warburg impedance to account for the diffusional contribution at low frequencies.^61^ The small semicircular contribution observed at high-to-intermediate frequencies is associated with the charge-transfer process, resulting in a charge-transfer resistance (R_CT1_) of approximately 657 Ω. This R_CT_ is enough to indicate favorable electron-transfer kinetics at the CarboInk surface, in agreement with the electrochemical behavior observed by cyclic voltammetry.

After Au electrodeposition, the Au/SPE exhibited a modified impedance response (Figure S2B), with an additional contribution associated with the presence of the metal on the sensor surface. The experimental data were fitted using the modified equivalent circuit shown in the inset (x^2^ = 1.37 × 10^–4^), which considers the different contributions of the carbon-based and Au-modified interfaces. Similar equivalent circuits have been reported for AuNP-modified carbon electrodes, where the Au modification introduces an additional interfacial contribution and decreases the charge-transfer resistance (R_CT1_ and _2_).^62^ The R_CT_ values obtained for the carbon-based layer and Au-modified interface were 418 Ω, and 0.815 Ω, respectively. The lower R_CT_ associated with the Au-modified interface indicates that the metal enhanced the charge-transfer process on the device surface. This behavior is consistent with the SEM results, which showed distinct Au-rich domains on the electrode surface. Table S2 comprises all the parameters associated with both equivalent circuits, such as the solution resistance (R_S_) and the constant phase elements’ admittance magnitudes (Y_0_) and exponential (n).

The Kramers–Kronig transformation test, commonly used to validate impedance data, was employed to validate the experimental results. The test consists of obtaining the real component of the data through the imaginary one and vice versa, if either is available at a given frequency. The calculated part is then fitted and correlated with the experimental results. Figure S2C shows the test results, with a x^2^ of complex impedance (Z) is 2.72 × 10^–5^, for the real part (Z′) is 1.29 × 10^–5^ and for the imaginary part, (−Z″), is 1.43 × 10^–5^. For the Au-modified device data, shown in Figure S2D, the x^2^ values are 4.68 × 10^–6^, 2.74 × 10^–6^ and 1.95 × 10^–6^, for Z, Z′ and -Z″, respectively. As usual, the lower the x^2^, more significant the data fit.^63^ Therefore, the presented values can be considered acceptable.

The knowledge of the properties and behaviors of this system, as is, was also necessary to make clear how the modifications of the surface would affect its NS1 detection capabilities. This protein cannot be determined, with enough sensitivity, without the addition of proper structural layers, that could provide sufficient signal alterations in the presence of the protein. To achieve it, the methodology of adding CYS and GA self-assembled layers over the Au structures was employed.^64,65^ To investigate the change in properties, cyclic voltammetry (Figure 6A) was performed in the presence of 1.0 mmol L^–1^ ferrocenemethanol, in 1.0 mmol L^–1^ phosphate buffer (pH 7.4), at v = 50 mV s^–1^. The addition of CYS barely changed both anodic and cathodic peaks, regarding their magnitude and peak potential: for Au/SPE, the I_anodic_ = 11.6 μA (E = 112 mV) and −I_Cathodic_ = 9.2 μA (E = 42 mV); while for CYS/Au/SPE the values are as follows: I_Anodic_ = 11.4 μA (E = 118 mV) and −I_Cathodic_ = 8.6 (E = 36 mV).

**Figure 6:**
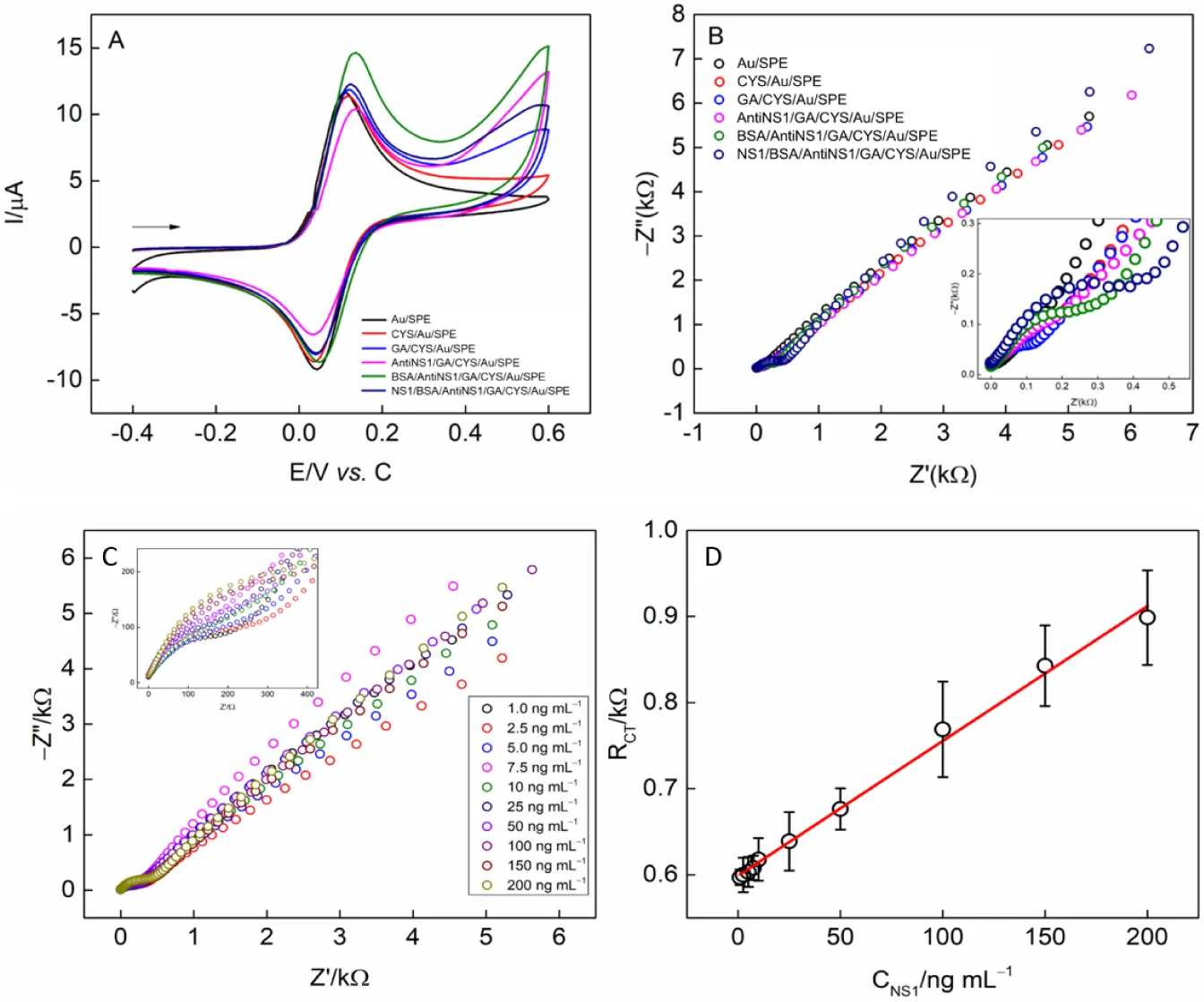
(A) Cyclic voltammograms and (B) Nyquist diagrams obtained by Au/SPE (**black**), CYS/Au/SPE (**red**), GA/CYS/Au/SPE (**blue**), AntiNS1/GA/CYS/Au/SPE (**pink**), BSA/AntiNS1/GA/CYS/Au/SPE (**green**) and NS1/BSA/AntiNS1/GA/CYS/Au/SPE (**navy**), in the presence of 1.0 mmol L^–1^ ferrocenemethanol and 1.0 mmol L^–1^ PB (pH 7.4); (A) v = 50 mV s^–1^; (B) inset shows a zoom of the resistance semicircles; E = 75 mV. (C) Nyquist diagrams obtained by NS1/BSA/AntiNS1/GA/CYS/Au/SPE after the interaction with different concentrations of NS1, in the presence of 1.0 mmol L^–1^ ferrocenemethanol, in 1.0 mmol L^–1^ PB (pH 7.4); E = 75 mV; (D) R_CT_ vs. C_NS1_ correlation.

When GA was added to the surface, the peaks remained mostly the same (I_Anodic_ = 11.9 μA (E = 122 mV) and −I_Cathodic_ = 8.1 (E = 40 mV)), but an increase in O_2_ generation was observed at higher potentials (400+ mV). This evolution can be noted in all following modifications, in different degrees. Afterward, the device was ready for the addition of a self-assembled antibody layer. AntiNS1 was therefore added to the system, causing significant changes to its behavior: the redox-pair has diminished magnitude, also becoming slightly more irreversible, with I_Anodic_ becoming 10.4 μA, at E = 136 mV, and −I_Cathodic_ being 8.6 μA, at E = 48 mV. To guarantee that the response obtained by the device is due to the interaction between antibody and antigen, bovine serum albumin was added to the surface, to saturate the nonspecific sites. At this moment, the redox pair magnitude is highly increased (I_Anodic_ = 14.6 μA, at E = 138 mV, and −I_Cathodic_ = 6.6 μA, at E = 34 mV), probably due to a catalytic effect that can be observed with BSA in the presence of some metals.^65–67^ When adding the target protein, NS1, it was expected that the signal would diminish, due to the protein acting as an insulator. This was experimentally proven, with I_Anodic_ decreasing to 12.3 μA (E = 126 mV), and −I_Cathodic_ changing to 7.9 μA (E = 42 mV).

Figure 6B presents the EIS data for the same modification process. The Au-modified system presented a R_CT_ of 20 Ω, with a barely perceptible resistance semicircle. The addition of CYS and GA changed the R_CT_ to 133 and 123 Ω, respectively, corroborating the CV data. After the formation of the AntiNS1 layer, the R_CT_ increased to 209 Ω, followed by a significant change to 308 Ω, caused by BSA. With the addition of NS1 and its interaction with AntiNS1, the R_CT_ further increased to 410 Ω, while also presenting a well-defined resistance phase semicircle.

After the proper modification, a calibration curve was obtained by modifying the device with known concentrations of NS1, and evaluating its R_CT_ in the presence of 1.0 mmol L^–1^ ferrocenemethanol, in 1.0 mmol L^–1^ PB (pH = 7.4), see Figure 6C and D. A linear behavior between the R_CT_ and the NS1 concentration was observed in the range of 1.0 to 200 ng mL^–1^, resulting in the calibration curve R_CT_ (kΩ) = 1.56 × C_NS1_ (ng mL^–1^) + 599, with R^2^ = 0.9956. The limit of detection (LOD) was calculated by the IUPAC method of 3 times the standard deviation of 10 blank analyses, divided by the slope, corresponding to the value of 0.34 ng mL^–1^. The concentration of dengue virus nonstructural protein 1 (NS1) in patient serum typically ranges from nanograms to micrograms per milliliter during the acute phase of infection and can be detected from the first day after fever onset up to approximately the ninth day.^68^ In some cases, NS1 levels may reach values as high as 50 μg mL^–1^. Although NS1 concentrations do not differ significantly between primary and secondary infections, secondary infections may present a faster decrease in free NS1 due to the formation of NS1–antibody immune complexes.^69^ Therefore, the low LOD obtained demonstrates that the proposed immunosensor is sufficiently sensitive for early detection, even in situations where circulating NS1 levels are reduced by immune complex formation.^70^

As this device is proposed as a multielectrode platform, it is important to evaluate the deviation between electrodes. Therefore, relative standard deviation (RSD) calculations were carried out considering repeatability and reproducibility among four electrodes per device, in the same experimental setup as the analytical curve, with each device modified with 50 ng mL^–1^ NS1. Considering data variance between measurements (repeatability, n = 4), electrode 1 presented RSD of 3.6%, electrode 2 of 3.4%, electrode 3 of 4.8% and electrode 4 of 4.0%. When considering the deviation of the same experiment among all electrodes (reproducibility, n = 4), the numbers were slightly higher: the calculated RSD was 5.8%. It is important to highlight that these RSD values are obtained in a complex process of competition between electrodes, and therefore are considered acceptable for such an application.

The device was then applied to the determination of NS1 in samples of human blood serum. Samples were prepared with spiked known concentrations of the target protein, and the devices were modified with 2.0 μL of these at the final stage. Table 2 presents all data obtained, in which it is possible to note that the recovery ranged from 96.6 to 115%, suggesting that the system is valid for applications in real and complex samples.

**Table 2:** Determination of NS1 Protein in Human Blood Serum Samples

Sample	Added (ng mL^–1^)	Detected (ng mL^–1^)	Recovery (%)
A	10.0	10.5 ± 0.4	105 ± 4
B	50.0	48.3 ± 1.5	96.6 ± 3.0
C	100	115 ± 4	115 ± 3
D	125	142 ± 4	113 ± 3
E	150	170 ± 5	114 ± 3
F	200	196 ± 7	97.9 ± 3.4

Although the device showed satisfactory analytical performance in spiked serum, broader analytical validation is still required, including cross-reactivity studies against related arboviral proteins, evaluation in naturally infected clinical samples, and storage stability assessment.

The selectivity of the immunosensor was evaluated by EIS using the developed AntiNS1/GA/CYS/AuCB platform in the presence of the target analyte (Dengue NS1, 150 ng mL^–1^), common endogenous electroactive species found in human serum, and a nontarget viral protein. Glucose (3.0 × 10^–2^ mol L^–1^, 5.40 mg mL^–1^) and dopamine (1.0 × 10^–4^ mol L^–1^, 0.015 mg mL^–1^) were selected as potential interferents and evaluated at their upper physiological concentrations to represent a worst-case interference scenario. In addition, the SARS-CoV-2 spike protein (150 ng mL^–1^) was included as a nontarget viral protein to investigate possible nonspecific recognition. As shown in Figure S3, a pronounced increase in the charge transfer resistance R_CT_ was observed only after incubation with Dengue NS1, whereas the responses obtained for glucose, dopamine, and the SARS-CoV-2 spike protein were negligible and remained close to the immunosensor baseline. These results demonstrate that common serum constituents and an unrelated viral protein do not significantly interfere with the analytical response, confirming the excellent selectivity of the proposed immunosensor toward Dengue NS1.

The stability of the immunosensor was first evaluated over five consecutive days using a sensor exposed to 150 ng mL^–1^ NS1. The response obtained immediately after sensor fabrication (Day 0), corresponding to the analytical calibration curve point, was used as the reference for comparison with subsequent measurements (Days 1–5). The Nyquist plots exhibited only minor variations throughout the evaluation period, indicating that the sensing interface remained stable during storage and repeated use. Accordingly, the charge-transfer resistance (Rct) values remained within the confidence interval shown in Figure S4, ranging from 815 to 889 Ω over the 5 days.

To further assess the practical applicability of the proposed immunosensor, its long-term storage stability was investigated over 30 days. Additional BSA/AntiNS1/GA/CYS/Au/SPE immunosensors fabricated in the same batch were stored at 4 °C and tested after 30 days. Before electrochemical measurements, the stored immunosensors were incubated with 150 ng mL^–1^ NS1 under the same experimental conditions used for freshly prepared devices. As shown in Figure S4, the charge-transfer resistance measured after 30 days remained within the confidence interval of the freshly prepared immunosensors, demonstrating that the biofunctionalized sensing interface preserved its analytical performance after prolonged refrigerated storage. These findings confirm the excellent storage stability of the proposed immunosensor and further support its suitability for practical applications.

Taking advantage of the data obtained during the stability study, the reproducibility of the fabrication process was also assessed using four independently prepared immunosensors monitored over 5 days (n = 20 measurements). The interelectrode reproducibility, calculated for each day, yielded relative standard deviation (RSD) values of 1.46%, 2.96%, 3.55%, 3.06%, and 1.36% for days 1–5, respectively. Considering all measurements, an average R_CT_ value of 851 Ω and a standard deviation of 25.6 Ω were obtained, resulting in an overall RSD of 3.01%. These low RSD values demonstrate the excellent reproducibility of the sensor fabrication procedure. Additionally, the repeatability of individual electrodes was evaluated throughout the 5 days. The calculated RSD values were 2.51%, 2.73%, 1.68%, and 3.94% for electrodes 1–4, respectively, confirming the consistency of the electrochemical response over time. The low variability observed among independently fabricated devices and during repeated measurements demonstrates the robustness and reliability of the proposed sensing platform. Overall, the results demonstrate that the proposed immunosensor exhibits high selectivity toward NS1 protein, excellent storage stability over at least 5 days, and satisfactory repeatability and reproducibility, with all RSD values remaining below 4.0%, which is considered suitable for practical analytical applications.

Afterward, the proposed immunosensor was benchmarked against previously reported NS1 detection platforms (Table 3). Compared to the epitope-imprinted MIP sensor reported by Sirivibulkovit et al.,^71^ the present device achieves an approximately 24-fold lower limit of detection (LOD) (0.34 vs 8.23 ng mL^–1^) while maintaining a label-free detection strategy. In addition, the linear working range extends toward lower NS1 concentrations, improving analytical performance within the early diagnostic window.

**Table 3:** Comparison between the Present Study and State-of-the-Art Published Works and the Proposed Sensor

Biosensor	Technique	LOD (ng mL^–1^)	Linear range (ng mL^–1^)	Reference
Au electrode modified with different aptamers	EIS	0.05	0.01 to 1000	^73^
GCE modified with Au and graphene materials^a^	DPV and SWV	1.43	0.01 to 100	^74^
		2.40		
AuSPE modified with epitope-imprinted polymers^b^	DPV	8.23	10 to 100	^71^
Lateral flow-based immunosensor	EIS	0.50	1.0 to 25	^72^
**SPE with 4 simultaneous Au-modified electrodes**	**EIS**	**0.34**	**1.0 to 200**	**This work**

a Glassy carbon electrode modified with biowaste-derived Au nanoparticles and reduced graphene oxide suspension.

b Commercial screen-printed devices made with gold-based ink.

In relation to the Lateral flow-based immunosensor developed by Sinawang et al.,^72^ the proposed sensor exhibits a slightly lower LOD (0.34 vs 0.50 ng mL^–1^), while offering a broader linear range extending up to 200 ng mL^–1^. This wider dynamic interval spans from the clinical positivity threshold to elevated NS1 levels, enabling reliable quantification across the full clinically relevant concentration range.

When compared with the ultrasensitive aptasensor fabricated on bulk gold substrates,^73^ the present platform trades part of the extreme sensitivity for enhanced practicality. Specifically, the use of a disposable, low-cost screen-printed architecture incorporating four working electrodes enables simultaneous measurements, internal controls, and potential multiplexed detection within a single device. This configuration significantly improves analytical robustness and applicability for point-of-care settings.

Parkhe et al.^74^ developed a glassy carbon electrode modified with biowaste-derived Au nanoparticles and reduced graphene oxide for label-free NS1 detection using DPV and SWV. Although it demonstrated good analytical performance, its LOD is higher than that achieved here. Moreover, the GCE-based design requires nanoparticle synthesis and nanocomposite assembly, making it more complex and less suitable for miniaturization than the disposable Au-modified SPE platform proposed in this work.

Overall, the proposed immunosensor demonstrates competitive analytical performance while offering practical advantages in terms of cost, disposability, and multielectrode capability, reinforcing its potential as a viable label-free alternative for dengue NS1 diagnostics.

## 4 Conclusion

This work describes the development of a multielectrode electrochemical immunosensor that was successfully applied toward the determination of dengue NS1 protein. This device contains four individually functioning electrodes, all printed with a commercially purchased Carboink. The four working electrodes were modified with Au nanoparticles, and this process was optimized using a central composite matrix. The optimized electrodeposition parameters significantly enhanced the electroactive surface area and electron transfer rate, while the functionalization with cysteamine, glutaraldehyde, and anti-NS1 antibodies ensured a successful biorecognition interface assembly. A linear range was obtained between 1.0 and 200 ng mL^–1^, with R^2^ of 0.9956. The device demonstrated a low detection limit (0.34 ng·mL^–1^), excellent linearity, and reproducible analytical performance, validating its applicability to a complex matrix (human serum). The mean repeatability RSD (n = 4) for each electrode was 4.1%, while the reproducibility RSD (n = 4) was 5.8%, cementing it as having an acceptable deviation between measurements and electrodes. Overall, its multielectrode platform nature allows for potential reinforcements on point-of-care tools in dengue diagnosis, with a promise of more accessible, accurate and rapid diagnostics.

## Supplementary Material





## Notes

Cyclic voltammetry and electrochemical impedance spectroscopy characterization of the screen-printed electrodes, including Kramers–Kronig validation and equivalent circuit fitting; evaluation of the immunosensor selectivity in the presence of potential interfering species; stability assessment of the immunosensor during storage at 4 °C; electrodeposition optimization parameters; and equivalent circuit fitting parameters for the bare and gold-modified screen-printed electrodes (PDF)
The preparation of the electrochemical sensor, its modification with gold nanoparticles, the fabrication of the NS1 biosensor, and the electrochemical detection procedure (MP4)
