# Supplementary material for: Gold-Enhanced Multielectrode Electrochemical Immunosensor for Rapid Electrochemical Detection of Dengue NS1
Source: ACS Omega. 2026 Oct 19;11(43):66295–307. doi: 10.1021/acsomega.6c04898 (PMC13737831; doi:10.1021/acsomega.6c04898)
Supplement: Supplementary file 1 [file ao6c04898_si_001.pdf]

Supplementary Material

Gold-Enhanced Multi-electrode Electrochemical Immunosensor for Rapid Electrochemical Detection of Dengue NS1

Luiz Otávio Orzari[a, b, c, d], *Bruna Silva Faria*[c, d], *Jéssica Rocha Camargo*[c, d], *Lucas Aguilera Mariano*[e, f], *Déborah Christine Azzi*[e, f], *Alexandre Gatti*[e, f], *Geisiany Maria de Queiroz*[e], *Bruno Campos Janegitz**[c, d]

[a] *Institute of Chemistry, State University of Campinas, Campinas, 13083-970 SP, Brazil*

[b] *Center for Innovation in New Energies, Campinas, 13083-841 SP, Brazil*

[c] *Laboratory of Sensors, Nanomedicine and Nanostructured Materials, Federal University of São Carlos, 13600-970, Araras, SP, Brazil*

[d] *Department of Nature Sciences, Mathematics and Education, Federal University of São Carlos, 13600-970, Araras, SP, Brazil*

[e] *Faculdade São Leopoldo Mandic, 13606-134, Araras- SP, Brazil*

f ADB Pesquisa e Desenvolvimento Ltda.

*Corresponding author: brunocj@ufscar.br

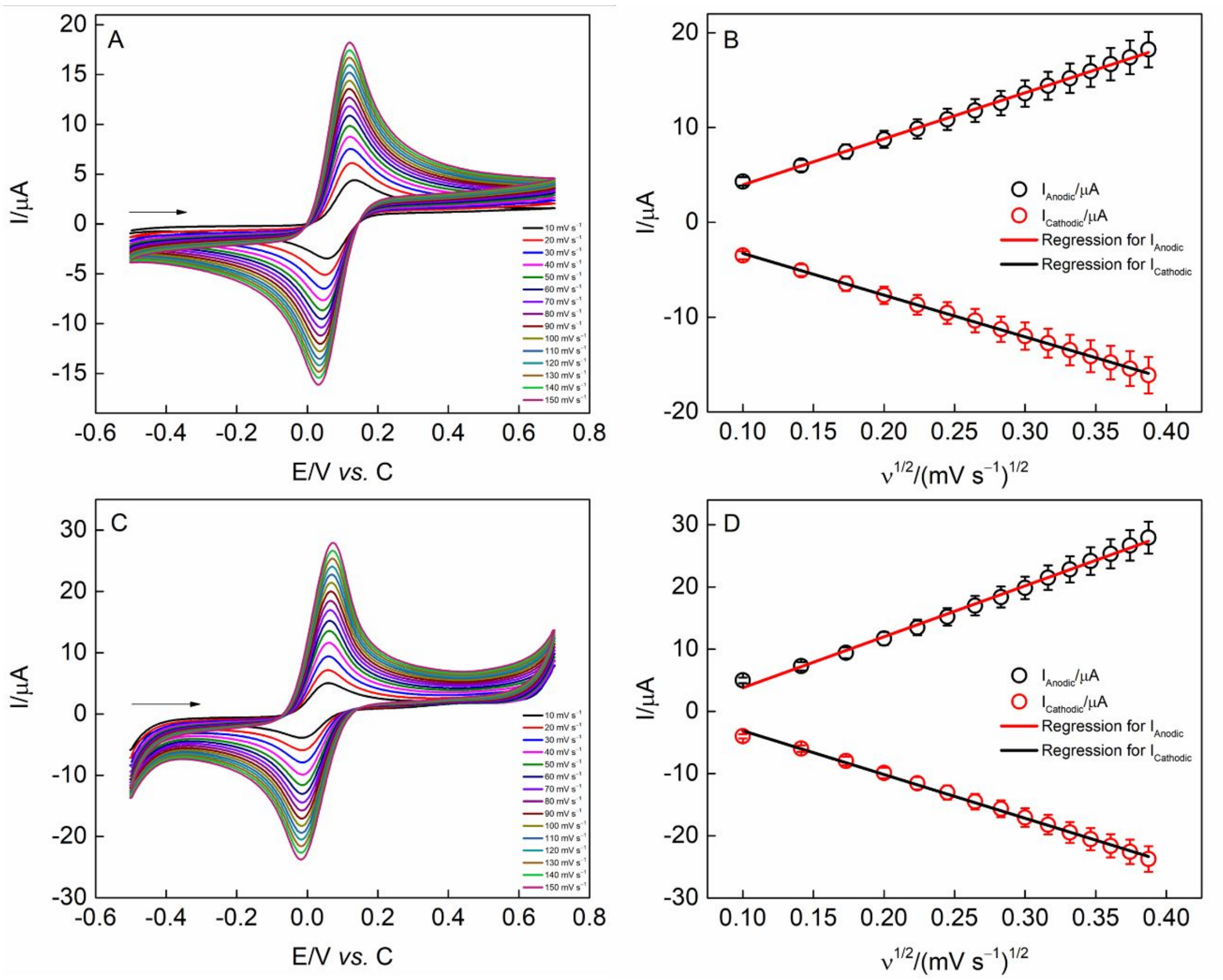

Figure S1. (A) Cyclic voltammograms obtained by CarboInk SPE in presence of 1.0 mmol L^{-1} ferrocenemethanol and 0.1 mol L^{-1} KCl with varying ν; (B) I *vs.* $\nu^{1/2}$ correlation for SPE; (C) Cyclic voltammograms obtained by Au/SPE in presence of 1.0 mmol L^{-1} ferrocenemethanol and 0.1 mol L^{-1} KCl with varying ν; (D) I *vs.* $\nu^{1/2}$ correlation for Au/SPE

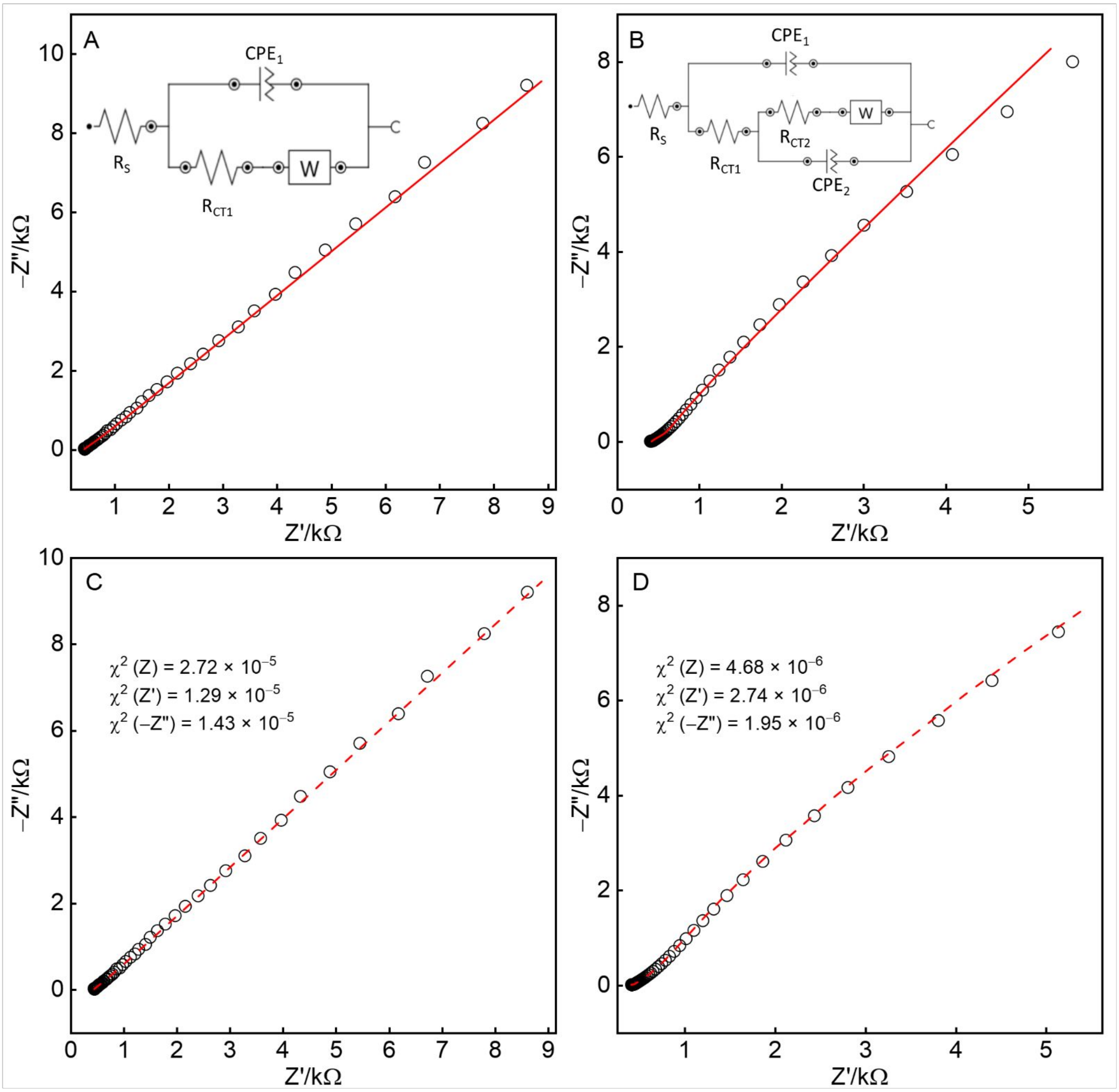

Figure S2. Nyquist diagrams obtained by (A) CarboInk SPE and (B) Au/SPE in the presence of 1.0 mmol L^{-1} ferrocenemethanol, in 0.1 mol L^{-1} KCl; E = 0.05 V. C and D are the Kramers-Kronig evaluation of A and B, respectively. Open circles represent the experimental data, and solid lines represent the corresponding fitted curves. Insets show the equivalent electrical circuits used for the fitting of the impedance data.

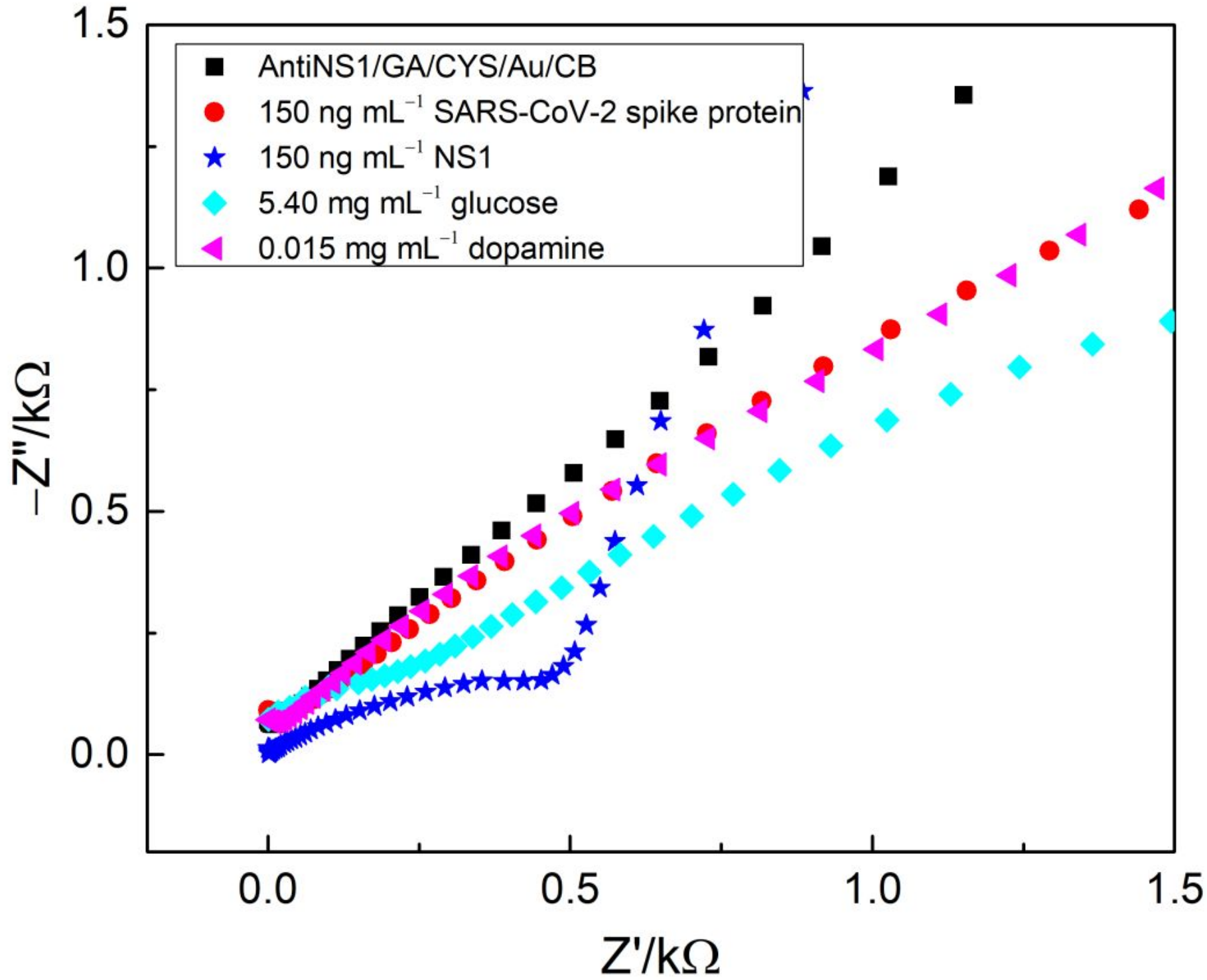

Figure S3. Nyquist plots obtained with the AntiNS1/GA/CYS/Au/CB immunosensor in the presence of common serum constituents at their maximum physiological concentrations (glucose: 3.0×10^{-2} mol L^{-1}, 5.40 mg mL^{-1}; dopamine: 1.0×10^{-4} mol L^{-1}, 0.015 mg mL^{-1}) and a non-target protein (SARS-CoV-2 spike protein, 150 ng mL^{-1}), demonstrating the selectivity of the immunosensor toward dengue NS1. Electrochemical impedance spectroscopy measurements were performed in 1.0 mmol L^{-1} ferrocenemethanol prepared in 1.0 mmol L^{-1} phosphate buffer (PB, pH 7.4), E = 75 mV. The concentration of NS1 was 150 ng mL^{-1}.

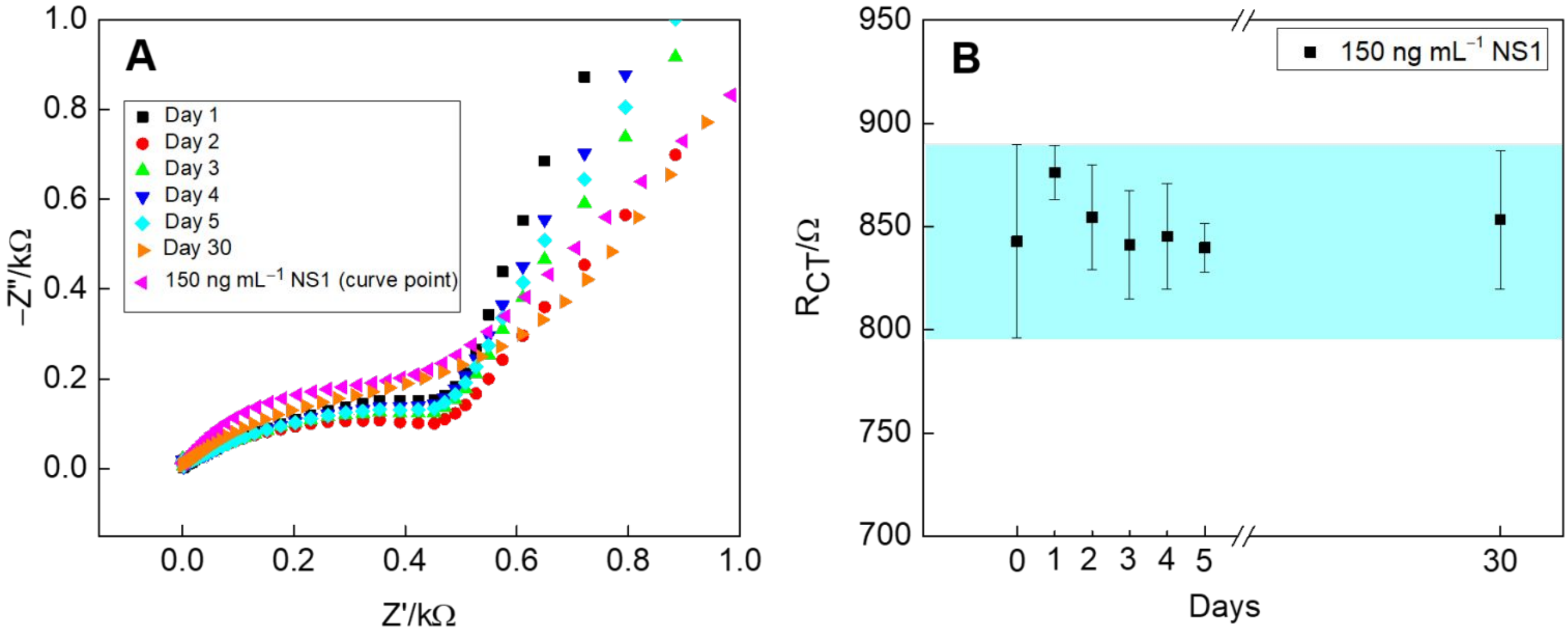

Figure S4. (A) Nyquist diagrams obtained for the NS1/BSA/AntiNS1/GA/CYS/Au/SPE immunosensor in the presence of 150 ng mL^{-1} NS1 during the stability evaluation. Day 0 corresponds to the analytical calibration curve point acquired immediately after sensor fabrication and used as the initial reference response. Day 1 to 5 and 30 correspond to immunosensors stored at 4 °C, with NS1 added only immediately before the electrochemical measurement. (B) Charge-transfer resistance (RCT) values recorded throughout the stability study; the shaded area indicates the confidence interval associated with the analytical response at 150 ng mL^{-1} NS1. Error bars correspond to the standard deviation of four WE (n = 4).

Table S1. Electrodeposition parameters investigated, their evaluated values and optimal values found

Parameter	Investigated values (−1 and +1)	Optimal value chosen
Electrodeposition time	30 and 300 s	300
Applied potential	−0.9 and −0.6 V	−0.8 V

Table S2. Equivalent circuit fitting values for CarboInk SPE and Au/SPE

Parameter	CarboInk SPE	Au/SPE
R_S (Ω)	439	400
CPE_1, Yo (S s^n)	5.94×10^{-5}	8.15×10^{-5}
n_1	0.561	0.596
R_{CT1} (Ω)	657	418
CPE_2, Yo (S s^n)	----	2.56×10^{-5}
n_2	----	0.832
R_{CT2} (Ω)	----	0.815
W (S s^5)	4.36×10^{-5}	1.27×10^{-9}
χ^2	2.77×10^{-4}	1.37×10^{-3}
